# Reduced Susceptibility to Praziquantel among Naturally Occurring Kenyan Isolates of *Schistosoma mansoni*

**DOI:** 10.1371/journal.pntd.0000504

**Published:** 2009-08-18

**Authors:** Sandra D. Melman, Michelle L. Steinauer, Charles Cunningham, Laura S. Kubatko, Ibrahim N. Mwangi, Nirvana Barker Wynn, Martin W. Mutuku, Diana M. S. Karanja, Daniel G. Colley, Carla L. Black, William Evan Secor, Gerald M. Mkoji, Eric S. Loker

**Affiliations:** 1 Center for Evolutionary and Theoretical Immunology, Department of Biology, University of New Mexico, Albuquerque, New Mexico, United States of America; 2 Departments of Statistics and Evolution, Ecology, and Organismal Biology, The Ohio State University, Columbus, Ohio, United States of America; 3 Centre for Biotechnology Research and Development, Kenya Medical Research Institute, Nairobi, Kenya; 4 Center for Global Health Research, Kenya Medical Research Institute, Kisumu, Kenya; 5 Center for Tropical and Emerging Global Diseases and Department of Microbiology, University of Georgia, Athens, Georgia, United States of America; 6 Centers for Disease Control and Prevention, Division of Parasitic Diseases, Atlanta, Georgia, United States of America; Australian Centre for International and Tropical Health, Australia

## Abstract

**Background:**

The near exclusive use of praziquantel (PZQ) for treatment of human schistosomiasis has raised concerns about the possible emergence of drug-resistant schistosomes.

**Methodology/Principal Findings:**

We measured susceptibility to PZQ of isolates of *Schistosoma mansoni* obtained from patients from Kisumu, Kenya continuously exposed to infection as a consequence of their occupations as car washers or sand harvesters. We used a) an *in vitro* assay with miracidia, b) an *in vivo* assay targeting adult worms in mice and c) an *in vitro* assay targeting adult schistosomes perfused from mice. In the miracidia assay, in which miracidia from human patients were exposed to PZQ *in vitro*, reduced susceptibility was associated with previous treatment of the patient with PZQ. One isolate (“KCW”) that was less susceptible to PZQ and had been derived from a patient who had never fully cured despite multiple treatments was studied further. In an *in vivo* assay of adult worms, the KCW isolate was significantly less susceptible to PZQ than two other isolates from natural infections in Kenya and two lab-reared strains of *S. mansoni*. The *in vitro* adult assay, based on measuring length changes of adults following exposure to and recovery from PZQ, confirmed that the KCW isolate was less susceptible to PZQ than the other isolates tested. A sub-isolate of KCW maintained separately and tested after three years was susceptible to PZQ, indicative that the trait of reduced sensitivity could be lost if selection was not maintained.

**Conclusions/Significance:**

Isolates of *S. mansoni* from some patients in Kisumu have lower susceptibility to PZQ, including one from a patient who was never fully cured after repeated rounds of treatment administered over several years. As use of PZQ continues, continued selection for worms with diminished susceptibility is possible, and the probability of emergence of resistance will increase as large reservoirs of untreated worms diminish. The potential for rapid emergence of resistance should be an important consideration of treatment programs.

## Author Summary

The emergence of drug resistant pathogens is a great challenge to the control of infectious diseases. Schistosomiasis is one of the world's greatest neglected tropical diseases, and it is primarily controlled with the drug praziquantel. This drug is often used by repeatedly treating patients to maintain reduced worm burdens, an ideal situation to encourage the evolution of resistant worms. Although drug based control programs are increasing, monitoring efforts for drug resistance remain rare. We measured drug susceptibility of schistosomes from a cohort of patients in Kenya who are enrolled in a longitudinal study in which they are repeatedly treated with praziquantel. We found that schistosomes from previously treated patients were significantly less susceptible than those that were not. Also, schistosomes derived from a single patient who had been treated with praziquantel 18 times showed marked resistance. Although the findings of this study indicated that reduced drug susceptibility occurs in this population of schistosomes, this trait does not seem to be spreading widely or creating clinical levels of resistance. We hypothesize that the trait remains at low frequency because of the large population of schistosomes that are not exposed to the drug and/or potential fitness costs associated with reduced susceptibility.

## Introduction

Schistosomiasis is one of the most common human parasitic diseases in the world. An estimated total of 207 million persons are infected worldwide, 97% of which are on the African continent [1]. The chronic and debilitating nature of the disease results in high costs in public health and economic productivity in developing countries, and has prompted the initiation of large scale control programs [2]. *Schistosoma mansoni* is one of the most common etiological agents for human schistosomiasis, and is estimated to infect more than 83 million humans in 54 countries [3].

Praziquantel (PZQ) is the least expensive, easiest to use and most readily available of all currently available schistosomicides [4]. It is highly effective against all schistosome species that are known to infect humans and is well-tolerated, making it suitable for mass treatment campaigns. These campaigns are particularly targeted at school-age children who represent the most heavily infected segment of the population [5],[6]. Although such programs have immediate and significant salutary effects, three general concerns are that they 1) inevitably leave some individuals untreated; 2) some individuals are treated but left uncured; and 3) they do not interrupt transmission, making re-infection a reality [7]. Another major concern of all anthelminthic and antibiotic drugs is the potential for resistance to develop and spread throughout the population, making the drug useless for treatment and control. Even though PZQ efficacy is generally high, reported cure rates are variable ranging from 60 to 95% [8]–[10]. Potential explanations for incomplete cures are use of doses that are actually sub-curative in people, or the presence of drug resistance traits present in natural populations of worms.

The extensive use of PZQ for over 20 years in some African nations has raised concern regarding the selection of drug resistant worms [5], [11]–[13]. Artificial selection in the laboratory has produced resistant strains of *S. mansoni* in only 2 generations of repeated exposure to sub-lethal doses of the drug in mice [11], thus demonstrating that resistance is more than a hypothetical possibility. Low cure rates in response to PZQ in the field appeared 10–15 years after the beginning of its use on a mass scale in Egypt and after a recent introduction of the worms in Senegal [12]–[16]. In both of these cases, worms from the uncured patients were also less susceptible to PZQ when tested in a mouse model [17]. Therefore, traits of the worms themselves led to PZQ failure, although other factors were also suspected to contribute including host factors, heavy worm burdens, and pre-patent infections [13],[17]. Reports of difficulties in obtaining cures among travelers with schistosomiasis [18] further underscore the need to remain vigilant. Most published discussions of this topic conclude that convincing evidence for the clinically relevant emergence of PZQ resistance in the field is still lacking [4],[5],[16],[19],[20]. Once drug resistance reaches clinical relevance it becomes a difficult problem to solve. Therefore, vigilant monitoring aimed at the prevention of clinical drug resistance is critical to treatment and control of infectious diseases.

Measuring the impact of PZQ on adult worms harbored by human subjects is at best an indirect process in that it relies on the relatively insensitive method of measuring reduction in schistosome egg excretion in treated individuals [21]. Also, several factors influence PZQ's effects such as variable pharmacokinetics in different individuals, differences in immune responses to the worms, maturity of worms, and genetic variability of the worms themselves, both among individual hosts and geographic regions [13],[16],[20],[22],[23]. Obtaining an isolate from human patients for further *in vivo* tests in laboratory hosts such as mice can address some of these concerns [13],[17] and can be complemented with *in vitro* testing that removes host-induced effects. *In vitro* tests for PZQ sensitivity have been developed for use on schistosomes [24]–[26]. Within a given isolate, the susceptibility of miracidia correlates with the susceptibility of adult worms [26]. Using miracidia in these assays potentially allows the testing of schistosomes derived from a large number of human infections.

The aim of this study was to evaluate the level of susceptibility to PZQ among a natural population of *S. mansoni* that were derived from Kenyan car washers and sand harvesters occupationally exposed to schistosome infection in Lake Victoria. These people have been enrolled in a longitudinal study designed to investigate immunologically-based host resistance to *S. mansoni* re-infection, and many of the participants have been treated with PZQ on multiple occasions throughout the study [27]–[30]. We first used an *in vitro* assay with miracidia to evaluate PZQ susceptibility of *S. mansoni* from several patients. Then, an isolate of *S. mansoni* was established in laboratory hosts from one of the patients, who had never fully cured after initial or several subsequent PZQ treatments. The susceptibility of adult worms of this and other Kenyan isolates and of two laboratory stocks of *S. mansoni* were compared using both conventional *in vivo* trials in mice, and with an *in vitro* assay with adult worms.

## Materials and Methods

### Source of *S. mansoni* isolates from human subjects

The patient-derived *S. mansoni* isolates were recovered from eggs in the fecal samples of adult males working as car washers or sand harvesters in or near Kisumu, western Kenya. The car washers use the shallow water along the Lake Victoria shoreline to wash cars and trucks and so are repeatedly in contact with water in an area where snails infected with *S. mansoni* snails have been repeatedly found [31]. The sand harvesters also experience extensive contact with lake water as they shovel sand from the submerged lake bottom into boats, often exposed to water from the chest down for hours each day. Since June 1995, the car washers have been continuously enrolled in several longitudinal studies that evaluate resistance to *S. mansoni* infection [27]–[30]. The sand harvesters were enrolled in similar studies starting in March 2005. For each patient, information is available regarding intensity of infection and history of exposure to treatment with PZQ since the time of their enrollment in these studies. As part of the ongoing work associated with these studies, fecal samples derived from each patient are regularly tested for *S. mansoni* using the modified Kato-Katz technique. For the purposes of the present study, miracidia hatched from *S. mansoni* eggs were used either directly in the PZQ susceptibility assay for miracidia described below or, in two cases, were used to establish laboratory isolates.

### *S. mansoni* isolates propagated in the laboratory for further study

Miracidia obtained from eggs in positive fecal samples from individual patients were used to establish the following isolates in laboratory raised *B. sudanica* and six to eight week old outbred mice:

*KCW*: The KCW isolate was established from a car washer who had received 18 PZQ treatments since the beginning of the study. Following initial and all subsequent treatments, egg counts in this patient had never fallen to zero [28], suggesting the responsiveness of worms from this patient to PZQ was worthy of further study. At the second generation, this isolate was divided and maintained as separate “sub-isolates” in New Mexico and in Kenya. Worms of the New Mexico sub-isolate were used in the experiments below unless otherwise noted.

*KSH*: The KSH isolate was established from a sand harvester who had never been treated with PZQ prior to this study.

*KAS*: This isolate was established from a human fecal sample collected near a stream (Asao) about 38 Km south east from Kisumu in 2006 (−0.33256°S, 34.99914°E).

*KNY*: The KNY isolate was established from cercariae obtained from *B. sudanica* collected in Nyabera marsh (−0.10971°S, 34.77461°E) on the outskirts of Kisumu on the road to Ahero. Both KAS and KNY isolates were from areas not previously included in PZQ treatment campaigns of local school children.

In addition to the isolates obtained from Kenya patients, the following isolates were used for comparison:

*PR1*: This laboratory stock is originally of Puerto Rican origin. It has been maintained in the laboratory, including at the University of New Mexico, for more than 20 years.

*NMRI*: This laboratory stock also originated from Puerto Rico and has been maintained at the Biomedical Research Institute, Rockville, Maryland (www.afbr-bri.com).

### *In vitro* assay to ascertain susceptibility of miracidia to PZQ

To test the idea that miracidia derived directly from different patients may differ in their susceptibility to PZQ, particularly given that some patients (see KCW above) had never fully cured following treatment with PZQ, we employed a modified version of the *in vitro* technique developed by Liang et al. [26]. Freshly hatched miracidia from the stools of sand harvesters (n = 14) and car washers (n = 9) were placed in each well (3–6 miracidia in each well) in a 96-well plate in 40 µl of aged tap water. Each row of the plate (one group of miracidia) received a different concentration of PZQ: 0 M (control), 10^−5^ M, or 10^−6^ M, in a 40 µl volume. PZQ was prepared as a stock solution of 10^−4^ M in 1% DMSO, and the final concentration of DMSO was 0.1% in all wells including the control wells. The mean number of groups of miracidia used per patient per concentration of PZQ was 10.2 (range: 4–12) (dependent on the number of miracidia obtained from a fecal sample). This design was repeated with miracidia from three different fecal samples from each patient. Miracidia were observed with the aid of a dissecting microscope prior to, 10 min and 20 min after addition of PZQ, and the observer had no knowledge of the PZQ concentration of each well. The number of miracidia that were alive and dead in each well was recorded. Miracidia were considered dead if they remained immobile. We present only results using 10^−5^ M at the 20 min observation time to simplify the presentation and because they are representative of the combinations of observation times (10 or 20 minutes) and PZQ concentrations (10^−5^ M, or 10^−6^ M) used. A Generalized Estimating Equations (GEE) approach was used to fit a logistic regression model to the data using the Proc GENMOD procedure in SAS 9.1. Separate models were fit for each concentration of PZQ (0 M, 10^−6^ M and 10^−5^ M). The number of previous PZQ treatments the subjects received was included in the model using an indicator variable that was set to ‘1’ if a patient had received no previous treatments, and‘0’ for patients who had received one or more previous treatments, and the time of exposure to the drug *in vitro* (0, 10 or 20 min).

### *In vivo* assay to ascertain susceptibility of adult worms to PZQ

For each isolate of *S. mansoni* examined, 10 to 30 outbred mice were infected with 200 cercariae per mouse. Seven and a half weeks after exposure, mice were randomly divided into two groups, one of which was given 1000 mg/kg PZQ dissolved in 2% Cremaphor EL over 4 consecutive days (250 mg/kg per day). This dose was chosen because it will achieve a parasite reduction of at least 95% in mice [32]. The other group was given only the vehicle (2% Cremaphor EL) as a control. Two weeks after treatment, mice from both groups were euthanized and perfused with RPMI medium [33]. The body cavity, liver, and mesenteric veins were examined for worms after perfusion to ensure all worms were found. This assay was repeated two more times with subsequent generations of the KCW isolate, three more times for the PR1 isolate, and once more for both the NMRI and KNY isolates. We were unable to perpetuate the KAS isolate due to difficulties in hatching the eggs obtained from infected mice and to establish snail infections, making further experiments with it impossible.

To eliminate the possibility that low PZQ susceptibility was due to a longer development time of the KCW worms, an additional *in vivo* assay was performed in which treatment was given at two time points. Mice were exposed to *S. mansoni*, randomly divided into 3 groups, and treatment was administered at either 7.5 or 10.5 weeks after exposure. The control group received the sham treatment at 7.5 weeks post exposure. Immature worms are not susceptible to PZQ, and studies of the NMRI isolate indicated that susceptibility corresponds to the onset of reproductive development at 6 to 7 weeks post infection [34].

The sub-isolate of KCW that was kept at the Kenya Medical Research Institute (KEMRI) was tested for PZQ sensitivity *in vivo* after 3 years of passages in the absence of drug pressure or testing. Mice infected with this subset of KCW were treated at 10 weeks post-exposure, following the above protocol.

The efficacy of treatment (ET) with PZQ as applied to the different isolates was measured as the percent of reduction of worm burdens based on the numbers recovered using the formula [35]:
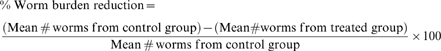



The number of worms recovered from the treated and control groups were compared across *S. mansoni* isolates with a randomized Generalized Linear Model 2-way ANOVA after log transformation of worm counts with SAS 9.1. The main factors considered were *S. mansoni* isolate, and treatment; 2-way interactions among the main factors were analyzed.

### *In vitro* assay to ascertain susceptibility of adult worms to PZQ

To complement the *in vivo* mouse treatment results an *in vitro* assay was devised to assess susceptibility of adult worms of *S. mansoni* to PZQ. Adult worms exposed to PZQ immediately contract. Although the mechanism by which PZQ causes this contraction is not fully understood, it is accompanied by a rapid influx of calcium ions, a slower influx of sodium ions and a decreased influx of potassium ions [36]. The rationale of our assay was to use the degree of contraction and subsequent recovery from contraction as a measure of PZQ susceptibility. We compared the length of the worms before exposure, during exposure (when the worms are contracted), and after a 24 hour recovery period to allow surviving worms to regain their normal lengths. Survival rates were also determined.

Adult male worms (KCW, KNY and PR1, and the sub-isolate of KCW maintained in Kenya) were recovered by perfusion of mice and incubated overnight, at 37°C in an atmosphere of 5% CO_2_, in RPMI medium supplemented with 20% Fetal Bovine Serum, 100–500 IU Penicillin, and 100 µg/ml Streptomycin. Overnight incubation allowed for recovery from the stress caused by the perfusion. A single worm was placed in each well of a 96-well plate in 180 µl of supplemented RPMI medium. Before exposure to PZQ, each worm was photographed with a Nikon Coolpix 4500 camera mounted on a dissecting microscope phototube with a Thales Optem digital camera adapter. Then, 20 µl of either the control medium or a PZQ solution, of five different concentrations, was added to each well, resulting in final concentrations of 0, 0.8, 8.0, 80.0, 400.0, and 800.0 µg/ml. The control solution contained 0.1% DMSO, the same concentration as that in the experimental wells. The worms were incubated in the PZQ solution at 37°C for 3 hours and then photographed. The medium in each well was removed; the worms were washed three times, and were then transferred to a new 96-well plate in fresh RPMI medium, and held at 37°C. Each worm was re-photographed at the end of the 24 hour recovery period.

The images of the worms before treatment and after recovery were processed with Metamorph v.4.65 software using the “fiber length” option which measures the object's length. The length of the worms was compared across *S. mansoni* isolates and PZQ concentrations and analyzed statistically with SAS 9.1 with a randomized ANOVA design; this was followed by Tukey's multiple comparisons to find significant pair-wise differences.

This research project has been reviewed and approved by the University of New Mexico's Institutional Animal Care and Use Committee (IACUC), and the institutional review boards of the University of Georgia and the Centers for Disease Control and Prevention, the Scientific Steering Committee of the Kenya Medical Research Institute, and the KEMRI/National Ethics Review Board of Kenya. All investigators/assistants in this study have attained animal use certification regarding the ethical treatment of animals.

## Results

### *In vitro* effects of PZQ on miracidia derived from different patients

**Figure 1:**
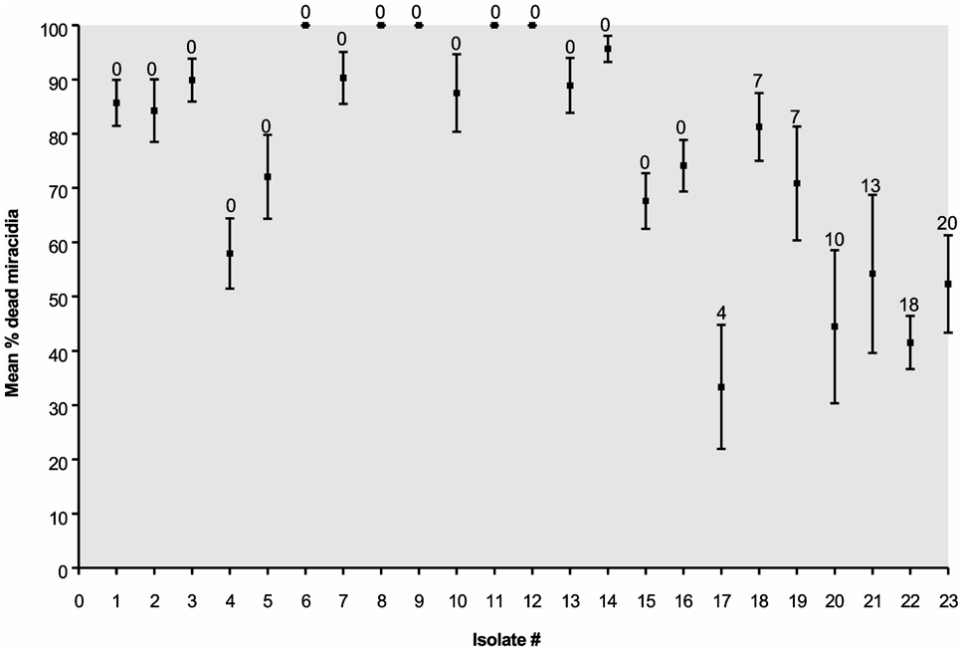
*In vitro* efficacy of 10^−5^ M PZQ in killing *S. mansoni* miracidia after 20 minutes of exposure in 96 well plates. Miracidia were hatched from fecal samples collected from human patients from the Kisumu area in Kenya, and the assay run in triplicate. The percentage of miracidia dead at the end of the 20 min observation period is graphed. Error bars correspond to standard error of the mean. Numbers above bars correspond to number of PZQ treatments. Data for parasites obtained from sand harvesters are indicated by squares and circles for car washers.

As expected, the *in vitro* effect of PZQ on the survival odds of miracidia was significantly influenced by drug concentration and time of exposure. To simplify data presentation, we show the results of exposure of miracidia derived from 23 different patients to 10^−5^ M PZQ after 20 minutes (Figure 1). For all time points examined, all miracidia held in water without PZQ continued to swim normally. There was considerable variation among the miracidia derived from different patients with respect to susceptibility to PZQ. The logistic regression model indicated that the odds of survival of miracidia in the *in vitro* assay upon exposure to 10^−5^ M PZQ are increased among those from patients who had received previous treatment compared to those from patients who had never been treated. Specifically, we obtained an estimated odds ratio of 2.42 [with a corresponding 95% confidence interval of (1.94, 3.01)] for comparing survival of miracidia in a previously-treated versus untreated group. In other words, the odds of surviving PZQ exposure were 2.42 times higher for miracidia collected from individuals who were previously treated than for those collected from patients who had never before been treated.

Miracidia obtained from the car washer who had never fully cured following treatment and from whom the KCW isolate was derived were among the least susceptible to PZQ (# 22, Figure 1). We then passaged an isolate from a patient who had never been treated (KSH) and the KCW isolate through snails and mice in the lab and tested miracidia from the mice to determine their PZQ susceptibility. KCW miracidia were significantly less susceptible (p<0.05) to PZQ (11% miracidia killed, from 6 trials) than KSH miracidia (75% miracidia killed, from 2 trials), suggesting that the reduced PZQ susceptibility of KCW was a heritable trait.

### *In vivo* effects of PZQ on adult worms in mouse infections

With subsequent mouse infections we compared the PZQ susceptibility of adults of the KCW isolate with adults from other Kenyan isolates (KAS and KNY) or from long-maintained laboratory stocks (PR1 and NMRI).

**Figure 2:**
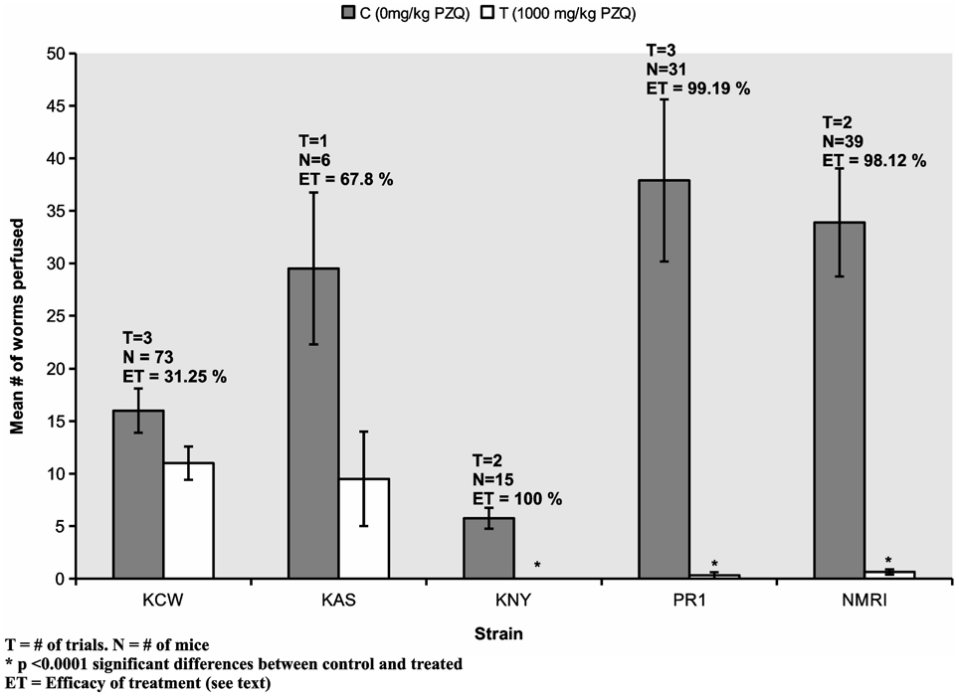
Average number of *S. mansoni* adult worms perfused from mice, following treatment with 1000 mg/kg of PZQ in Cremaphor 2% EL. Treatment was administered at 7.5 weeks p.i., and mice perfused 2 weeks after treatment. Efficacy of treatment was calculated as described in text. The p values for the comparison between mean # of worms recovered from control versus treated groups were as follows: KCW, p = 0.072; the sample size for KAS was too small to assess statistical significance; KNY, p<0.0001; PR1, p<0.0001; NMRI, p<0.0001. Error bars correspond to standard error of the mean.

The results (Figure 2) are summarized based on multiple independent trials performed on successive generations of the worms, except for KAS, for which we had data from only one trial. The worm yields obtained from mice varied among isolates, which is not surprising given that the Kenyan isolates were recently derived from humans whereas the PR1 and NMRI isolates have been maintained in mice for decades. However, by reference to worm recoveries from corresponding untreated controls, we concluded that the efficacy of treatment was significantly lower in mice infected with KCW (31.3%) than in mice infected with *S. mansoni* from the other 4 sources (over 98% for PR1, NMRI, and KNY and 67.8% for KAS; p<0.05), using a randomized ANOVA design. It should be noted that while the sex ratio for the worms recovered from mice infected with the KCW strain was male biased, the infections were not predominantly single sexed.

Due to low infectivity for snails and mice and the inevitable attendant loss of genetic diversity, KAS worms recovered from mice were mostly or only males. The relatively low efficacy of treatment in mice infected with the KAS isolate (67.8%) might be explained by this phenomenon, since worms from single-sex infections are less susceptible to PZQ than those from mixed-sex infections [24].

The results of the *in vivo* trial to examine the effects of delayed maturation showed that the efficacy of treatment was 28.1% at 7.5 weeks post-exposure and 26.3% at 10.5 weeks post-exposure. The mean number of worms recovered from control and treated mice did not differ significantly at either 7.5 weeks post-exposure (control 32.0±7.9, treated 23.0±6.0, p = 0.39) or 10.5 weeks post-exposure (control 38.7±4.3, treated 28.2±8.1, p = 0.29). Worms from untreated control mice at both times were sexually mature, and the livers from the mice harboring these infections contained many granulomas. These results indicate that the reduced susceptibility to PZQ observed *in vivo* with the KCW isolate was not a result of delayed maturation rate of KCW worms.

### *In vitro* effects of PZQ on adult worm length and survival

**Figure 3:**
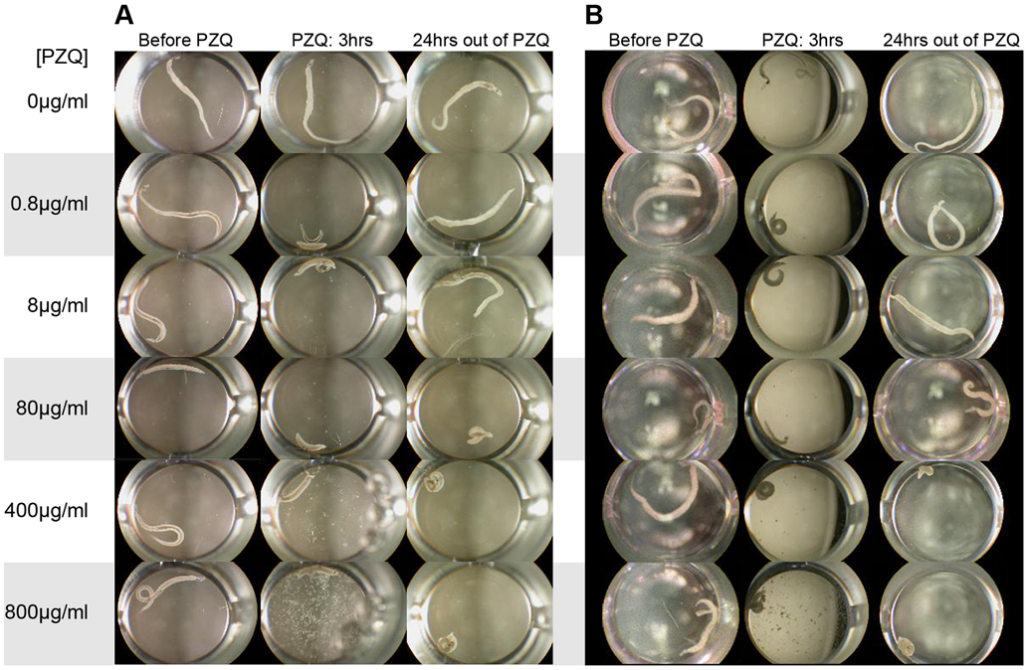
Photographs of *S. mansoni* adult worms of KNY (plate A) and KCW isolates (plate B), before exposure to PZQ (left column), after 3 hrs of continuous exposure to PZQ (central column), and 24 hrs after removal from PZQ. Concentration of PZQ is shown on the left side of the figure. The photographs show the effect of the drug on the length of the worms and the degree of recovery of the worm's length after removal from the sub-lethal concentrations of the drug. Note the difference in the response of the two isolates to the 8 µg/ml dose. The results for the PR1 strain which was also tested were similar to those shown for the KNY isolate.

In the *in vitro* assay to test adult worm PZQ susceptibility (Figure 3) we found that for all isolates, control worms not exposed to PZQ remained elongated at all observation times and there were no significant differences in worm length found among the three isolates (p = 0.678). Following exposure to PZQ, regardless of the dose administered, worms from all three isolates contracted. However, for worms that were treated with 80 µg/ml of PZQ, there were differences among isolates in the extent that they remained contracted after the 24 hour recovery period. KNY and PR1 remained fully contracted and did not move. These worms were considered dead as per the definition of Pica-Mattoccia and Cioli [24]. In contrast, KCW worms partially elongated and were moving and active, and had increased their body lengths significantly more than KNY or PR1 worms (p = 0.038). Worms of all three isolates recovered their normal length if they had been exposed to concentrations of PZQ lower than 80 µg/ml. Conversely, doses higher than 80 µg/ml of PZQ were lethal for all isolates.

### Stability of the PZQ insensitive trait

**Figure 4:**
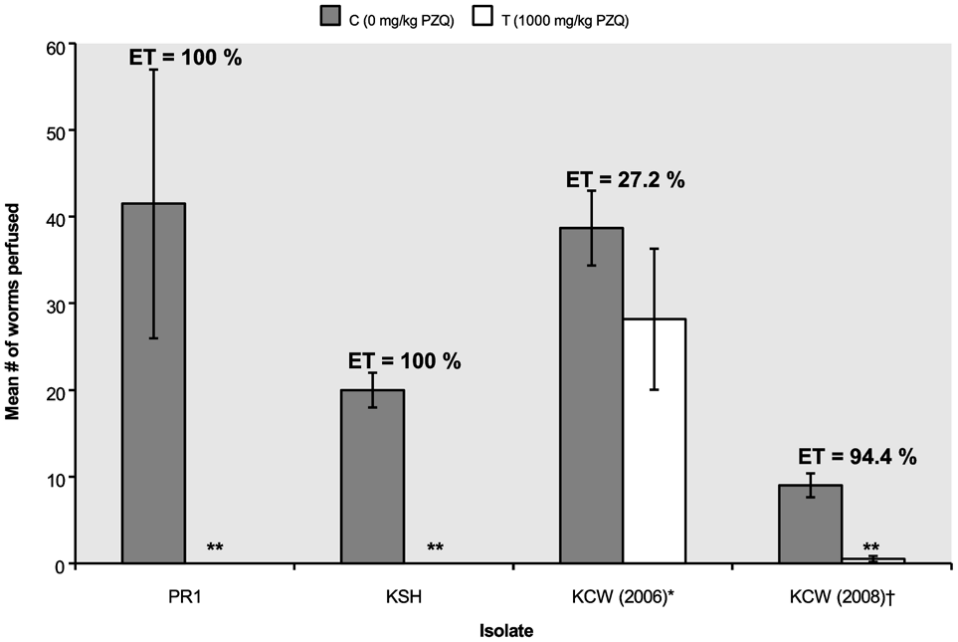
*In vivo s*usceptibility to PZQ of adult worms. KCW (2008) refers to a subset of the KCW isolate kept in Kenya since 2005 that was tested in 2008, after 3 years of passage in laboratory mice and snails in Kenya. KCW (2006) refers to a subset of the KCW isolate brought to New Mexico in 2005 right after isolation from the patient, and that was tested in this assay in 2006. Also shown are results obtained with the KSH isolate and the PR1 lab strain. ^**^Difference between average number of worms recovered from perfusion from control animals versus treated animal significant (α = 0.05). ET = Efficacy of treatment, was calculated as described in text.

After the initial *in vitro* assay of miracidia that indicated reduced susceptibility of the KCW isolate, additional miracidia from this patient were divided into two sub-isolates in 2005, one was maintained in New Mexico and one in Kenya. After 8 generations of passaging the Kenyan sub-isolate through mice and snails, we performed the three susceptibility assays and found that the trait for reduced susceptibility had been lost. Before splitting the KCW isolate, in the *in vitro* assay of miracidia the mean percentage of dead miracidia recorded was 11%, which was significantly lower than after 8 generations (98%) and for all other isolates tested: 87%, 79%, 100%, and 78% for the PR1, NMRI, KAS and KSH isolates, respectively (p<0.05). As shown in Figure 4, the subset of KCW maintained for eight passages over three years in Kenya (labeled KCW (2008)) showed no diminished susceptibility to PZQ in the *in vivo* trials as compared to PR1 or KSH, and was significantly more susceptible to PZQ than the New Mexico subset of KCW (labeled KCW (2006) to indicate the year it was tested). The *in vitro* test of adult worms showed similar results. The difference in mean body length after the 24 hour recovery period between the New Mexico KCW sub-isolate and PR1, KNY and Kenyan KCW (2008) was significant (p = 0.038). Kenyan KCW (2008), PR1 and KNY did not differ in this regard (p = 0.211). In combination, these results indicate that the KCW sub-isolate maintained in Kenya lost the trait of diminished sensitivity to PZQ that we saw in miracidia when KCW was first isolated, and in comparison to the New Mexico sub-isolate.

## Discussion

In this study, we investigated PZQ susceptibility of *S. mansoni* from a high-transmission endemic area [28],[31] using *in vitro* assays involving both miracidia and adult worms, and an *in vivo* assay measuring drug susceptibility of adult schistosomes. In this population, we found worms with reduced susceptibility to PZQ. Miracidia collected from multiple patients varied significantly in their susceptibility to PZQ, and this susceptibility was associated with whether the patient had a history of exposure to the drug. Miracidia from car washers were more tolerant to PZQ than those from sand harvesters, which is not surprising because the car washer focus had been studied since 1995, while the sand harvesters were just being recruited in the study and had no prior history of exposure to PZQ. These results may suggest population level differences; however, we note that the sites are relatively close (within 5 km of each other) and our ongoing population genetics studies suggest no population subdivision between these groups or any other schistosomes collected throughout the Kenyan portion of Lake Victoria [22]. We hypothesize that treatment of individual patients with PZQ led to the accumulation of resistant worms within a single host rather than PZQ exposure inducing resistance within a patient, although induction of hycanthone resistance in schistosomes has been reported previously [37].

More in-depth studies were undertaken on an isolate (KCW) established from a patient who yielded miracidia with relatively low susceptibility to PZQ. Both *in vivo* and *in vitro* assays of adult worms confirmed this observation and showed that they had significantly lower susceptibility to PZQ than adults from other recent Kenyan isolates or from lab stocks.

Drug resistance has evolved in many different microbial pathogens and parasites [38],[39], and is a looming concern for control of any pathogen, including schistosomes. Vigilant monitoring efforts are critical for preventing the spread and controlling the problem of drug resistance. The *in vitro* assays applied here were developed to monitor PZQ susceptibility of schistosomes, and have been validated by demonstrating for a variety of isolates that *in vitro* susceptibility of eggs, miracidia and cercariae to PZQ correlate with *in vivo* susceptibility of adult worms [26]. Such an *in vitro* assay is helpful given the inherent problems with measuring PZQ efficacy in humans, which is determined by measuring egg counts in fecal samples before and after treatment. Such an indirect measurement of efficacy can lead to erroneous conclusions about susceptibility of the schistosomes harbored by the treated person: they may harbor immature worms not susceptible to PZQ [16],[34], re-infections are likely in endemic areas and may confound interpretation of cure rates, and even though egg counts have diminished they may still harbor mature adult worms with temporarily inhibited egg production [40]. For such reasons, *in vitro* assays to determine drug susceptibility can be very useful, but they pose difficulties of their own. Most notably in our study was the difficulty of hatching eggs from field-collected fecal samples which made it hard to perform *in vitro* assays on populations of miracidia from some patients. Persistent difficulty in getting eggs to hatch ultimately led to the loss of the KCW sub-isolate maintained in New Mexico. Egg hatchability is an understudied phenomenon that could play an important role in the epidemiology of *S. mansoni* particularly if low hatching rates are an associated cost of drug resistance.

Inclusion in our study of other Kenyan isolates that proved to be susceptible to PZQ provides evidence of variable susceptibility in natural populations and strengthens our findings of reduced susceptibility because the laboratory stocks potentially could have abnormal responses to praziquantel. Interestingly, the intensity of infection achieved with the Kenyan isolates was lower than that resulting from laboratory stocks. This difference likely is due to host adaptation as the laboratory stocks have been maintained in mice for several generations. It is unlikely that intensity influenced the outcome of the *in vivo* trials as efficacy of treatment was comparable across replicates regardless of intensity. In clinical reports, high intensity sometimes correlates with reduced efficacy of PZQ, but this could be due to high recruitment rates and the presence of immature worms within the host, or merely be a consequence of the fact that complete cures, as measured by cessation of egg production, are harder to obtain in people with high worm burdens even though the efficacy of treatment is the same. Even if reduced efficacy with high intensity infections was a phenomenon relevant to our *in vivo* trials, it would strengthen our conclusions because our more susceptible worms were present in higher intensities.

Worm maturation is a potential alternative explanation for our results if the KCW worms develop more slowly in mice such that they were still immature and thus less susceptible to PZQ at standard treatment times. Although we saw no consistent evidence of a slower development rate, and KCW worms recovered upon perfusion at 7.5 weeks post-infection were mature, we nonetheless addressed this possibility in an experiment that allowed three weeks of additional development before treatment was administered. The low PZQ susceptibility of these older, fully mature worms renders any argument invoking slower development times an unlikely explanation for the low PZQ susceptibility of KCW worms.

In our study, diminished susceptibility to PZQ was a heritable trait that persisted across multiple generations: the KCW sub-isolate maintained in New Mexico retained reduced PZQ susceptibility over 6 generations, even in the absence of drug pressure. However, for the KCW sub-isolate maintained in Kenya, this trait was lost sometime during 8 generations of laboratory rearing. Loss of diminished susceptibility traits has also been reported for Egyptian field isolates brought into the laboratory [41]. The relative ease with which the trait of reduced PZQ susceptibility was lost may be associated with a fitness cost such as diminished asexual reproductive capacity in snails [41]. Fitness costs have been noted in other drug resistant organisms and play a large role in their evolution [42]. It is also interesting that with our divided isolates of KCW, the drug tolerant sub-isolate perished, but the susceptible sub-isolate persisted.

The data from this study suggest that at least some members of the *S. mansoni* population in the Lake Victoria region have lower susceptibility to PZQ. As with most anthelmithic resistance, it is assumed that this trait has occurred naturally within the population even prior to drug treatment in the region. In fact, the patient from whom the KCW isolate was derived was never fully cured, even after the very first treatment. Furthermore, a longitudinal epidemiological study of these patients over the course of 12 years reported an average cure rate of 66%, but no evidence of increased treatment failure or clinically relevant level of resistance over the course of the study [28]. These findings suggest that worms with reduced susceptibility to praziquantel occur in the population, and are more common in patients that have been repeatedly treated, but this trait remains in relatively low frequency throughout the entire worm population. We suggest two hypotheses for this observation. First, although drug pressure is strong on the select target group of patients in Kisumu, the overall population of *S. mansoni* in snails, humans and other mammals in the Lake Victoria basin is vast [31], leaving a large refugium of untreated worms that are not subject to selection. Therefore, after treatment, the frequency of resistance alleles would remain low in the remaining worm population and likely in subsequent generations. The size of the untreated refugium has been shown to play an important role controlling the spread of resistance to other anthelminthics [43]. A second factor is that there may be a fitness cost associated with resistance, which would also work to prevent accumulation of the trait in the population unless the benefits of resistance outweighed the costs [41]. This reality, one that likely applies in many schistosomiasis endemic areas in Africa where treatment programs are underway, lessens current concerns about the spread of PZQ-resistance. However, as treatment expands to an ever larger proportion of infected individuals, then the refugia for unexposed parasites will diminish in size, increasing the chances for resistance to emerge.

One of the most concerted programs of PZQ treatment, directed against *S. haematobium* in coastal Kenya, found variability in responsiveness to the drug, but no evidence of progressive emergence of PZQ resistance over an 8 year interval [19]. However, this program also involved a large refugium of untreated schistosomes because treatment was targeted to school children, and as the authors note, the limited coverage of the program may have reduced the tendency for resistance to spread. Based on computer modeling, it was predicted that emergence of PZQ resistance in this region should be anticipated within 10 to 20 years of its continued massive use [19].

Three aspects pertaining to the potential emergence of PZQ resistance are particularly deserving of additional study. The first is to determine if repeated cycles of infection and treatment of a single patient can favor the accumulation of a subset of *S. mansoni* adults more tolerant to PZQ and through recombination of alleles due to sexual reproduction, can lead to enhanced resistance in offspring. The pattern of decreased responsiveness to PZQ of miracidia derived from patients that had received multiple treatments, including KCW, is compatible with such a possibility. Trials to assess the safety and efficacy of higher doses for patients that fail to cure following exposure to standard PZQ dosages, or approval of more widespread availability of alternative treatments, such as oxamniquine, may both be prudent considerations when treatment failures occur. Second, because we lack a fundamental understanding of PZQ's mode of action, we are also ignorant of the natural variability in PZQ's targets in schistosome populations in endemic areas. It will be important to determine if variants inherently less susceptible to PZQ's effects are particularly likely to occur in the large, genetically diverse populations of species like *S. mansoni* [23] that still thrive throughout much of sub-Saharan Africa. More explicit knowledge of PZQ's targets will help us to devise much-needed improved assays for monitoring the emergence of resistance. Third, methods for genotyping individual eggs or miracidia need to be used to determine if the worm populations harbored by people before and after treatment show evidence of strong similarity, suggestive of the retention of fully adult worms that are not killed but merely temporarily silenced by PZQ treatment. Some Kisumu patients that have been treated remain negative for egg passage for long periods indicating they have been successfully cured and may even have acquired effective resistance to reinfection [30], but others resume egg production after treatment, indicative of acquisition of new worms, or possibly reactivation of existing worms.

We conclude by noting that, ironically, the maintenance of PZQ susceptibility may come at the expense of poor coverage and continued high transmission. Continued monitoring of PZQ susceptibility using assays such as the ones employed here, and new and improved assays, is warranted as increased use of PZQ in control programs becomes a reality.
